# Flexible and Electroactive Ionogel Graphene Composite Actuator

**DOI:** 10.3390/ma13030656

**Published:** 2020-02-01

**Authors:** Chao Lu, Xi Chen

**Affiliations:** 1 Department of Earth and Environmental Engineering, Columbia University, New York, NY 10027, USA; cl3865@columbia.edu; 2 School of Chemical Engineering, Northwest University, Xi’an 710069, China

**Keywords:** flexible material, ionogel graphene composite, electroactive actuator

## Abstract

Electrochemical actuators have attracted tremendous attention worldwide because of their critical significance to artificial intelligence. The development of electrochemical actuators—with the merits of low driven-voltage, lightweight, flexibility and large deformation—is an urgent task in the development of smart technologies. Nanomaterials with special structures and superior properties provide the opportunity for the development and application of smart actuators. Here, we report an electrochemical actuator based on an ionogel graphene composite, which is assembled with simple casting methodology and can be driven with a low voltage of 2.5 V. The flexible sandwich-structured actuator operates under a capacitive mechanism based on asymmetrical volume expansion of active ions under electrical stimulus. It shows a high specific capacitance of 39 F g^−1^ at current density of 1 A g^−1^ under potential of 2.5 V. The specific capacitance is calculated on the weight of graphene. The device presents a large actuation peak-to-peak displacement of 24 mm at a frequency of 0.1 Hz under the stimulus potential of 2.5 V, and it can still reach a large value of 12 mm at a high frequency of 1 Hz. The free length of the device is 25 mm. Notably, the device exhibits excellent air-working stability at frequency of 1 Hz under 2.5 V with the actuation displacement retention of 98%, even after 10,000 cycles. This study presents insights into the design of smart actuators based on nanomaterials, and will accelerate the development of artificial intelligence.

## 1. Introduction

Flexible electrochemical actuators are emerging as promising candidates for applications in smart scenarios—including in virtual reality, the internet of things and soft robotics—and have recently attracted tremendous research enthusiasm worldwide [1,2,3]. Electrochemical actuators are composed of one polyelectrolyte layer laminated with two electrode layers. MXene is a good candidate electrode material for actuators, because the interlayer spacing of MXene can be changed via the intercalation and de-intercalation of many kinds of ion, such as K^+^, Mg^2+^, Na^+^ [4]. The development of soft actuators with lightweight, low driven-voltage and large deformation is still kept a challenge, because conventional bulk structural materials, such as noble metals and bulk inorganic materials, cannot address these issues with limited structural properties [5,6,7]. In addition, aqueous electrolyte-based polyelectrolytes limit the working potential window, and can cause drawback effects and water loss problems when in actuation states [8,9,10]. Thus, it is urgent to develop high-performance electrochemical actuators with novel, material-based components.

With the rapid development of nanomaterials in recent decades, there now exists a great opportunity for the preparation of high-performance flexible electrochemical actuators based on advanced nanomaterials, such as graphene [11,12], graphitic carbon nitride [13,14], metal-organic frameworks [15,16], carbide derived carbon [17], carbon aerogel [18], and other template-synthesized nanomaterials [19,20]. These nanomaterials with large surface areas, special electronic structures and highly porous structures are potentially suitable for the fabrication of electrodes. The drawbacks induced by conventional aqueous electrolytes can be avoided using ionogel electrolytes, which are composed with ionic liquid and polymers [21,22]. Ionic liquid-based electrolytes—with the characteristics of a high boiling point, nonflammability, and chemical stability—can effectively prevent the actuators from water loss, solvent effects and drawback effects. Thus, it is a promising strategy to combine advantages of ionogel electrolytes with nanomaterial electrodes for the preparation of next-generation electrochemical actuators.

Here, we report a flexible and electroactive actuator based on graphene materials and ionogel electrolytes, which is fabricated through a simple solution casting method. Other previous works mostly utilized the hot-pressing method, which required sophisticated equipment and precise control of conditions [23,24,25]. Graphene is selected because of its high electronic conductivity, large surface area and porous structures, which can facilitate electron and ion transfer during actuation processes. A polyvinylidene fluoride/1-ethyl-3-methylimidazolium tetrafluoroborate (PVdF/EMIBF_4_) ionogel electrolyte was applied for its high ionic conductivity and wide potential window. The actuator shows the high specific capacitance of 39 F g^−1^ at current density of 1 A g^−1^ under potential of 2.5 V, indicating its high accommodation for active ions. The driven voltage of the actuator is only as low as 2.5 V, which is easily achievable using the dry batteries used in everyday life and thus presents great potential for artificial intelligence applications. The device also displays a large actuation displacement of 24 mm at frequency of 0.1 Hz under 2.5 V, and the displacement retains as high as 12 mm at higher frequency of 1 Hz. It is found that the actuator shows excellent cycling stability with high displacement retention of 98%, even over 10000 cycles at 1 Hz under 2.5 V. This study provides a promising strategy for the fabrication of high-performance flexible electrochemical actuators, and will also shed light on the design of other flexible electrochemical devices.

## 2. Materials and Methods

### 2.1. Materials

PVdF polymer and DMF (Dimethyl Formamide) solvent were obtained from Sinopharm Chemical Reagent Co., Ltd., (Shanghai, China). EMIBF_4_ was purchased from Millipore Sigma company (New York City, NY, USA). Graphene powder (NO: XFGNP009) was obtained from XFNANO Materials Tech Co., Ltd., (Nanjing, Jiangsu, China)

### 2.2. Synthesis of Ionogel Electrolyte

Firstly, 1.0 g PVdF and 1.5 g EMIBF_4_ were mixed in 30 mL DMF solvent at 30 °C. Then, the solution was poured into a mould and kept at 80 °C for 24 h. Finally, the PVdF/EMIBF_4_ electrolyte membrane was obtained by peeling off the mould after vacuum drying at 80 °C for 12 h.

### 2.3. Fabrication of Ionogel Graphene Composite Actuator

Firstly, 50 mg graphene powder and 5 mg PVdF were dispersed in 10 mL DMF for 1 h through horn sonication in ice bath. Subsequently, the suspension was cast onto one side of PVdF/EMIBF_4_ electrolyte and then dried at 80 °C for 12 h. Following that, the actuator was obtained by casting the other side with graphene electrode with same method. The thickness of the actuator is 117 μm.

### 2.4. Characterizations

SEM and TEM images were made with Hitachi S-4800 (New York City, NY, USA) and FEI Tecnai F200 (New York City, NY, USA), respectively. Electrochemical performances of ionic actuator were measured with Bio-logic Potentiostat VMP3 work station (Bio-Logic, Seyssinet-Pariset, France). The CV curve was measured in the potential range of 0–2.5 V with scan rate of 100 mV s^−1^. Electrochemical impedance spectroscopy was conducted from 10 mHz to 100 kHz with a voltage amplitude of 10 mV. Charge-discharge curve was conducted at 1 A g^−1^ under voltage of 2.5 V. The displacement of actuator was measured by Keyence LK-G800 laser positioning equipment (Keyence company, Osaka, Japan).

## 3. Results and Discussion

**Figure 1:**
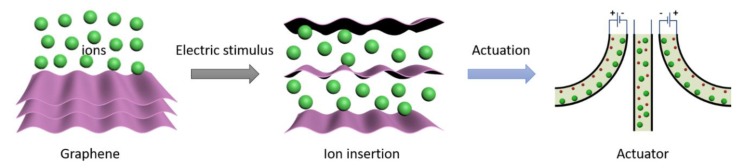
Schematic for mechanism of ionogel graphene composite actuator.

The mechanism and sandwich structure of ionogel graphene composite actuator are briefly illustrated in Figure 1. Typically, cations and anions migrate and insert into cathode and anode, respectively, under an applied electrical field. The insertion of ions induces the expansion of electrodes, because the layer distance of graphene enlarges with inserted ions. The cations and anions in ionogel electrolytes have different sizes, and thus will induce the asymmetric expansion of cathode and anode, which directly cause deformation of electrochemical actuators [5,25]. The as-described mechanism is based on a capacitive mechanism, and the actuation effect is mainly affected by accommodated ions in the electrodes. Nanomaterials with porous structures are promising for achieving large deformation in actuators. 

**Figure 2:**
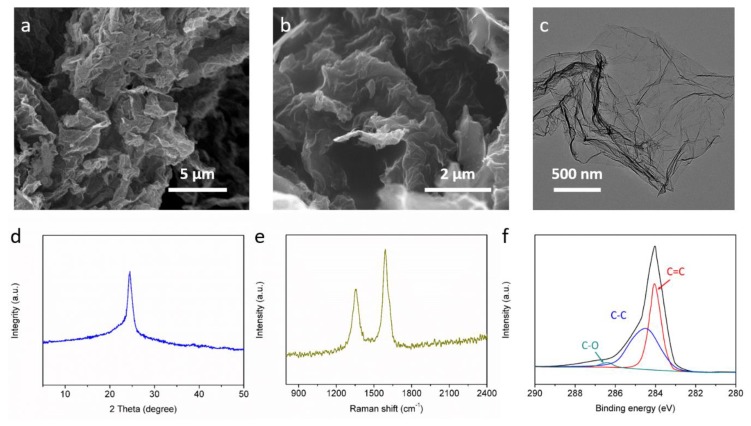
SEM (**a**,**b**) and TEM images (**c**) of graphene materials. (**d**–**f**) XRD, Raman and XPS spectras.

**Figure 3:**
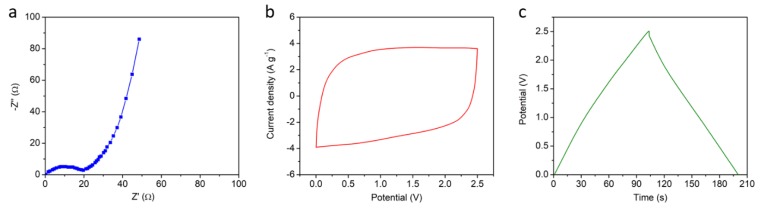
Electrochemical impedance spectroscopy spectra (**a**) of the actuator. Cyclic voltammetry curve (**b**) of the device at scan rate of 100 mV s^–1^. Charge-discharge curve (**c**) of the device at current density of 1 A g^–1^.

The morphology of graphene materials is presented in Figure 2, which has been investigated with scanning electron microscopy (SEM) and transmission electron microscopy (TEM) techniques. The SEM images in Figure 2a,b clearly verify the porous structure of graphene materials, which is favorable for accommodation of active ions migrated into electrodes. The TEM image in Figure 2c shows the typical two-dimensional layered structure of graphene materials. In the XRD spectra, the characteristic peak at around 25° is attributed to (002) diffraction of graphene. In the raman spectra, the peaks at 1350 and 1582 cm^−1^ are attributed to D (disordered and defective structure) and G (graphitic structure) bands of graphene. The XPS C 1*s* spectra can be fitted into three peaks, which are attributed to characterize the peaks of C–C, C=C and C–O. As illustrated by the scheme in Figure 1, the two-dimensional structure can promote ion immigration under an electrical field through the formation of layered ion channels for fast orientation transfer. In order to verify the potential of graphene materials for electrochemical actuators, a flexible actuator has been fabricated utilizing graphene materials as electrodes, and ionogel PVdF/EMIBF_4_ as an electrolyte. The electrochemical impedance spectroscopy (EIS) result is shown in Figure 3a, and it was found that the equivalent series resistance is only 19 Ω, which indicates good internal contact in the actuator. The cyclic voltammetry (CV) curve in Figure 3b displays a regular rectangle shape with the typical capacitive behavior of ion storage. The charge–discharge (CD) curve of the actuator at a current density of 1 A g^−1^ under a potential of 2.5 V is shown in Figure 3c. The near triangle shape of the curve verifies the high coulombic efficiency of the device under charge and discharge states, which is believed to promote electro-mechanical energy conversion in actuator.

**Figure 4:**
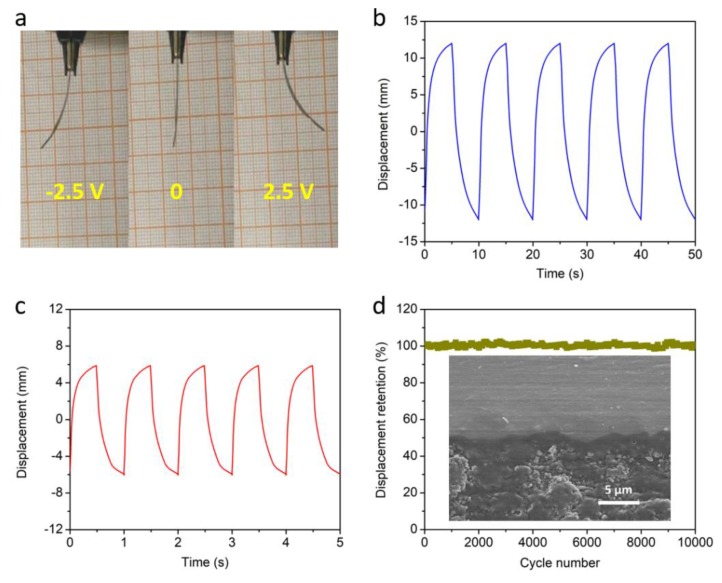
Optical images (**a**) of actuator under actuation states. Actuation displacements of actuator under potential of 2.5 V at frequencies of 0.1 Hz (**b**) and 1 Hz (**c**), respectively. Cycling stability of actuator (**d**) under potential of 2.5 V at frequency of 1 Hz over 10000 cycles. Inset is cross-section SEM image of device.

The flexible bimorph cantilever actuator is evaluated through multi-step voltage stimulus in Figure 4a. The deformation direction of the actuator can be precisely controlled by changing the electric property of the applied voltages. The actuator presents large actuation displacement of 24 mm at a frequency of 0.1 Hz under a potential of 2.5 V and operates stably in the first few cycles, as shown in Figure 4b. The actuation amplitude of the device can be adjusted through the control of applied frequencies. As displayed in Figure 4c, the displacement of the actuator decreases from 24 mm to 12 mm when the frequency increases from 0.1 Hz to 1 Hz. Even the frequency increases to a high level, the device also shows a negligible response time and keeps a high actuation deformation. As the cycling life of actuators is critical to their practical application, the cycling stability of an actuator has been evaluated at frequency of 1 Hz under potential of 2.5 V in Figure 4d. It is worth mentioning that the device shows excellent cycling performance with displacement retention as high as 98%, even after 10,000 actuation cycles. Compared with conventional noble metal electrode-based actuators, the graphene-based actuator in this study shows better cycling performances [5,26]. This is because noble metal electrodes usually have bulky and dense structures, which are easy to degrade under repeated working cycles. As a cross-section of the composite is important, we provide the cross-section SEM image of the actuator in the inset of Figure 4d. It is found that there is a tight coupling interface between the electrode and electrolyte layers, which guarantees the stable actuation performances of the device. The large and controllable actuation performance of this electrochemical actuator is a significant achievement in the smart actuation field, and should somewhat accelerate the development of artificial intelligence.

## 4. Conclusions

In summary, a flexible and electroactive ionogel graphene composite actuator has been prepared based on graphene electrodes and ionogel electrolytes through a simple solution casting method. The porous structure of graphene facilitates the accommodation of active ions, and the chemically stable ionogel electrolyte promotes the cycling performances of the device. The as-prepared actuator displays a high specific capacity of 39 F g^−1^ at a current density of 1 A a^−1^, under a voltage of 2.5 V. Moreover, the actuator can operate under a low driven voltage of 2.5 V, which can be easily realized using the dry batteries used in everyday life. It shows large deformations both at high and low actuation frequencies, and the deformation amplitude and direction can be adjusted by controlling the driven voltages. In addition, the device displays excellent cycling stability, with actuation retention of 98% over 10,000 cycles in an air atmosphere, indicating promising practical applications. The research conducted in designing in this work will provide insights for the design of other flexible electrochemical devices with long cycling lives.
